# Self-Reported Habitual Daily Physical Activity as an Independent Predictor of Coronary Artery Disease Extension in Patients with Myocardial Infarction: A Prospective Observational Study

**DOI:** 10.3390/jcm15103814

**Published:** 2026-05-15

**Authors:** Corina Cinezan, Maria Luiza Hiceag

**Affiliations:** 1 Department of Medical Disciplines, Faculty of Medicine and Pharmacy, University of Oradea, 410073 Oradea, Romania; 2 Clinical County Emergency Hospital Bihor, 410169 Oradea, Romania; 3 Cardiology Department, Rehabilitation Hospital, 400347 Cluj-Napoca, Romania; 4 Cardiology Department, Municipal Hospital Aiud, 515200 Aiud, Romania

**Keywords:** physical activity assessment, coronary angiography, multivessel disease, cardiovascular epidemiology, observational study, risk stratification

## Abstract

**Background**: The extent of coronary artery disease (CAD) is a major determinant of prognosis in patients with myocardial infarction (MI). While structured exercise is known to be cardioprotective, the association between habitual daily physical activity and angiographic CAD extension remains insufficiently characterized. **Methods**: In this prospective observational study, 269 patients were hospitalized with acute MI underwent coronary angiography. Habitual daily physical activity during the four weeks preceding admission was assessed using 10-point self-reported daily preadmission effort questions to help the patients to report a final effort score. CAD extension was classified as single-, double- or triple-vessel disease. Differences in daily effort across CAD categories were evaluated using the Kruskal–Wallis test. Independent predictors of CAD extension were identified using ordinal logistic regression adjusted for age, sex, smoking, hypertension, diabetes mellitus, hyperlipidemia and body mass index. **Results**: Daily preadmission effort decreased progressively with increasing CAD severity (mean scores: 7.44 in single-vessel, 4.93 in double-vessel and 3.69 in triple-vessel disease; *p* < 0.0001). In multivariable ordinal logistic regression analysis, older age, hypertension, diabetes mellitus and hyperlipidemia were independently associated with greater CAD extension. Higher daily preadmission effort was strongly and independently associated with lower CAD severity; each one-point increase in effort score was associated with a 46% reduction in the odds of more extensive CAD (odds ratio 0.54, 95% confidence interval 0.45–0.64; *p* < 0.0001). **Conclusions**: Greater habitual daily physical activity prior to myocardial infarction is independently associated with less extensive coronary artery disease. Assessment of daily preadmission effort may provide clinically useful information regarding coronary disease burden and highlights the potential importance of everyday physical activity in cardiovascular prevention. These findings should be interpreted with caution given the use of a non-validated, self-reported measure of physical activity and the observational study design.

## 1. Introduction

CAD is the leading cause of morbidity and mortality worldwide. MI, one of its most severe clinical manifestations, mostly results from the rupture of atherosclerotic plaques within the coronary arteries, resulting in severe narrowing or occlusion of the implied vessel [1,2,3]. This process constitutes the pathological mechanism of MI. Approximately 64% of acute coronary syndromes occur due to this process, followed by platelet-rich thrombosis [4]. Vulnerable plaques typically have thin fibrous caps (50–65 μm), large lipid cores and abundant inflammatory cells [5]. Upon rupture, the plaque releases thrombogenic contents, causing platelet activation and thrombus formation. Alternative mechanisms include plaque erosion (accounting for approximately 25% of cases) and calcific nodules [6].

Beyond the occurrence of MI itself, the angiographic extent of CAD represents a key determinant of prognosis, influencing long-term survival, left ventricular function and revascularization strategy [7,8]. Identifying modifiable factors associated with greater CAD severity is therefore of major clinical importance. The 3.5-year composite risk of late revascularization, myocardial infarction and death ranges from 1.5% for patients without CAD to 15% for three-vessel/left main disease [9]. Hazard ratios for mortality increase progressively with CAD severity, from 1.28 for non-obstructive CAD to 4.41 for three-vessel/left main disease compared to no CAD. The angiographic severity of coronary stenosis (≥70% for non-left main disease, ≥50% for left main disease) has been used to define significant stenosis and guide revascularization strategy [10].

Among established cardiovascular risk modifiers, physical activity plays a central role in vascular health. Done regularly, it improves endothelial function, reduces systemic inflammation, lowers sympathetic tone at rest, enhances insulin sensitivity and favorably influences blood pressure and lipid metabolism [11,12,13]. These processes are associated with slower atherosclerotic progression and improved cardiovascular outcomes. While the benefits of structured exercise programs are well-documented, habitual daily physical activity, encompassing occupational, domestic and incidental movement has been less extensively studied, particularly in patients presenting with acute MI. Among myocardial infarction survivors, maintaining or increasing physical activity levels is associated with substantial reductions in recurrent cardiovascular events and mortality. Compared to remaining inactive, the risk of all-cause mortality is 50% lower in those who remained active, 45% lower in those who became active and 20% lower in those whose activity decreased [14,15,16,17,18]. In MI survivors specifically, maintaining high physical activity or increasing activity from before to after MI is associated with 27–39% lower mortality risk.

Assessment of physical activity in the acute-care setting is challenging. Standardized questionnaires, although validated, are often time-consuming and impractical during hospitalization for MI. Consequently, routine daily activity levels prior to admission are rarely evaluated, despite their potential relevance to coronary disease burden [19,20]. Simple self-rated ordinal scales of habitual activity have demonstrated utility in epidemiological studies by capturing activity gradients that reflect real-world behavior and cumulative physical effort.

We therefore developed a pragmatic 10-point self-reported scale to assess daily preadmission effort in patients hospitalized with MI. This tool was designed to rapidly estimate habitual physical activity during the weeks preceding the index event.

The aim of this prospective study was to evaluate the relationship between daily preadmission effort and the extent of coronary artery disease in patients with myocardial infarction and to determine whether habitual daily activity is independently associated with increasing CAD severity.

We hypothesized that lower levels of habitual daily physical activity prior to myocardial infarction are independently associated with greater angiographic coronary artery disease extension.

However, assessment of physical activity in acute clinical settings remains challenging, as most validated instruments are impractical during hospitalization and self-reported measures may introduce recall bias and misclassification.

## 2. Materials and Methods

### 2.1. Study Design and Setting

This study was designed as a prospective observational cohort study conducted at the County Clinical Emergency Hospital Bihor, Romania, Department of Cardiology, a tertiary-care center providing interventional cardiology services. A total of 269 patients admitted with acute MI was consecutively screened for eligibility. All patients underwent diagnostic coronary angiography during the index hospitalization, with percutaneous coronary intervention performed when clinically indicated.

This study was conducted in accordance with the Declaration of Helsinki. The study protocol was approved by the Ethics Committee of Clinical County Emergency Hospital Bihor, Oradea (protocol number 34667/19 November 2025). Informed consent was obtained from all participants included in the study.

### 2.2. Study Population

Between November 2025 and March 2026, a total of 269 consecutive patients aged 35–85 years admitted with acute MI was included in the study. MI was defined according to the Fourth Universal Definition of Myocardial Infarction, requiring a rise and/or fall in cardiac troponin values with at least one clinical feature of myocardial ischemia (ischemic symptoms, electrocardiographic changes or imaging evidence). This definition was used as an inclusion criterion to ensure standardized diagnosis across the study population.

### 2.3. Inclusion Criteria

- Age between 35 and 85 years;
- Hospital admission with acute MI, both ST-elevation and non-ST-elevation;
- Underwent diagnostic coronary angiography during index hospitalization;
- Ability to provide written informed consent and complete the admission questionnaire, including the daily preadmission effort scale.

### 2.4. Exclusion Criteria

- Prior coronary artery bypass grafting;
- Severe comorbid illness associated with life expectancy < 1 year;
- Inability to perform physical activity due to an acute or chronic illness which limited it, such as infectious or autoimmune disease, severe heart failure, severe valvular disease, uncontrolled hypertension, pulmonary hypertension, neoplasms, anemia, arthrosis, pulmonary disease, severe obesity, severe kidney failure;
- Inability to provide informed consent.

**Figure 1:**
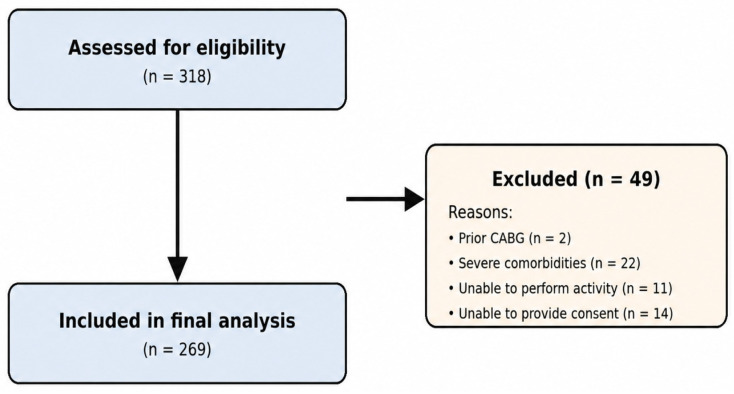
Study flow diagram. Flowchart illustrating patient selection, exclusion criteria and the final study population included in the analysis. CABG: coronary artery bypass grafting.

A total of 318 patients was assessed for eligibility, of whom 49 were excluded (two with prior coronary artery bypass grafting, 22 with severe comorbidities, 11 with inability to perform physical activities and 14 unable to provide informed consent). The final study population consisted of 269 patients. The study flow diagram is presented in Figure 1.

Although the study included a broad age range, age was modeled as a continuous variable to preserve statistical power. Future studies with larger sample sizes may allow stratified analyses to further explore potential age-specific effect.

### 2.5. Baseline Clinical Data Collection

Baseline demographic and clinical data were collected at admission and included the following:

- Age and sex;
- Smoking status (current smoker vs. non-smoker);
- Hypertension (documented history or use of antihypertensive medication);
- Diabetes mellitus (documented diagnosis or use of antidiabetic medication);
- Hyperlipidemia (documented diagnosis, lipid-lowering therapy, or total cholesterol > 200 mg/dL);
- Body mass index (BMI), calculated as weight (kg) divided by height squared (m^2^), in accordance with World Health Organization criteria [21].

### 2.6. Assessment of Daily Preadmission Effort

Habitual physical activity prior to hospital admission was assessed using a self-reported 10-point daily preadmission effort scale, administered within the first 24 h after admission for myocardial infarction. Patients were asked to retrospectively evaluate their average daily physical effort during the four weeks preceding the index event, taking into account all domains of activity, including occupational tasks, household activities, transportation and leisure-time movement.

The daily preadmission effort scale was established as a simple and pragmatic ordinal measure designed for feasibility in the acute-care setting, where detailed physical activity questionnaires are impractical. The extended questionnaire was used to facilitate a structured and comprehensive assessment of habitual physical activity across multiple domains, including occupational, domestic, and recreational activities, as well as perceived effort and functional capacity.

However, the quantitative variable used in the statistical analysis was the global effort score (1–10), which was self-reported by the patient based on their overall perception of daily physical activity. The individual questionnaire items were not summed up or weighted to generate the final score but were intended to support and standardize the patient’s self-assessment. This approach was chosen to preserve the simplicity and feasibility of the assessment in the acute clinical setting.

To improve the consistency of scoring, patients received verbal explanations and practical examples of low, moderate and high activity levels from trained study personnel. Patients were instructed to select the value that best represented their usual daily activity, rather than occasional or peak exertion. The scale was not intended to quantify precise energy expenditure but to capture relative gradients of habitual physical activity, consistent with its ordinal structure.

The scale was developed by the investigators based on conceptual frameworks used in epidemiological studies of physical activity, aiming to capture global habitual effort across multiple domains. It was not formally validated against objective measures such as accelerometry, nor was external validation performed prior to its use in this study. However, pilot testing was conducted in a small subset of patients (*n* = 23) to assess clarity and feasibility.

Intermediate scale levels were defined as follows:

- 1–2: Predominantly sedentary behavior.
- 3–4: Light daily activity (minimal walking, low occupational effort).
- 5–6: Moderate activity (regular walking, light physical work).
- 7–8: High activity (physically demanding daily routines).
- 9–10: Very high activity (intense manual labor or sustained exertion).

Data on pre-infarction symptoms (angina, dyspnea) were not systematically collected, which represents a limitation of the study.

The full questionnaire used to assess habitual physical activity, including all domains and the global effort score, is provided in Supplementary Materials S1.

### 2.7. Establishment of the Final Effort Score for Statistical Analysis

For each participant, the final daily preadmission effort score used in statistical analyses corresponded to the single integer value (1–10) selected by the patient at admission. No post hoc modification, weighting, transformation, or categorization of the score was applied.

Given that the scale reflects ordered levels of habitual physical activity, the effort score was treated as an ordinal variable. For descriptive and univariate analyses, effort scores were summarized using appropriate measures of central tendency and differences across coronary artery disease severity categories were evaluated using non-parametric statistical tests.

The daily preadmission effort score was categorized into tertiles (low [1–4], medium [5–7], high [8–10) based on its distribution in the study population. Stratified analyses were performed according to median age (≤median vs. >median) to evaluate the consistency of the association between effort and coronary artery disease (CAD) extent across age groups. Within each age stratum, differences in the distribution of effort categories across CAD extent were assessed using the chi-square test. A two-sided *p*-value < 0.05 was considered statistically significant.

For multivariable analysis, the daily preadmission effort score was entered into the ordinal logistic regression model as an ordinal predictor treated as quasi-continuous, under the assumption that each one-point increase represented a proportional increase in habitual physical activity. This approach preserved the full informational content of the scale and enabled assessment of a dose–response relationship between daily preadmission effort and coronary artery disease extension.

The global effort score used in the analysis was derived from the final item of the questionnaire.

### 2.8. Coronary Angiography and Classification of CAD Extension

All patients underwent diagnostic coronary angiography during hospitalization. Angiographic images were independently reviewed by two experienced interventional cardiologists who were blinded to patients’ daily effort scores.

Coronary artery disease extension was classified based on the number of major epicardial coronary arteries (left anterior descending, left circumflex, right coronary artery) demonstrating ≥70% luminal stenosis:

- Single-vessel disease: One major vessel affected.
- Double-vessel disease: Two major vessels affected.
- Triple-vessel disease: Three major vessels affected.

Patients with left main disease were excluded, due to the complexity of coronary artery lesions.

Discrepancies between reviewers were resolved by consensus. CAD extension was treated as an ordered categorical variable, reflecting increasing disease severity.

Several strategies were implemented to minimize bias. Recall bias was reduced by assessing physical activity within 24 h of hospital admission. Selection bias was limited through the inclusion of consecutive patients. Multivariable regression was used to adjust for major confounders including age, sex and cardiovascular risk factors.

### 2.9. Statistical Analysis

Continuous variables were summarized as means with standard deviations or medians with interquartile ranges, as appropriate. Categorical variables were expressed as counts and percentages. Differences in daily preadmission effort across CAD severity categories were assessed using the Kruskal–Wallis test.

Age was dichotomized according to the median value of the study population into two groups (≤median and >median) to perform stratified analyses. This approach was used to evaluate whether the association between preadmission effort and coronary artery disease (CAD) extent was consistent across different age strata.

To identify independent predictors of CAD extension, ordinal logistic regression (proportional odds model) was performed, with CAD severity (single-, double-, or triple-vessel disease) as the dependent variable. Covariates were selected based on clinical relevance and established associations with coronary artery disease. This model estimates the odds of being in a higher CAD severity category versus all lower categories combined. The proportional odds assumption was assessed and found to be acceptable.

Independent variables included age, sex, smoking status, hypertension, diabetes mellitus, hyperlipidemia, BMI and daily preadmission effort. Results are reported as odds ratios (ORs) with 95% confidence intervals (CIs). A two-sided *p*-value < 0.05 was considered statistically significant.

Sensitivity analyses were performed to assess the robustness of the primary findings. Specifically, the daily preadmission effort score was additionally modeled as a categorical variable, and the consistency of the association with coronary artery disease severity was evaluated.

All analyses were performed using Python (version 3.11) with the pandas, scipy and statsmodels libraries.

Given the exploratory nature of the study, a formal sample size calculation was not performed. However, the sample size of 269 patients was considered adequate based on the rule of at least 10 events per variable included in the regression model.

There were no missing data for the variables included in the analysis.

## 3. Results

### 3.1. Study Population

A total of 269 patients admitted with acute myocardial infarction was included in the analysis. The mean age of the study population was 60 ± 12 years (range 35–85 years) and 134 patients (49.8%) were male. The mean body mass index (BMI) was 27 ± 4 kg/m^2^.

Regarding cardiovascular risk factors, 121 patients (45.0%) were current smokers, 108 (40.1%) had a history of hypertension, 67 (24.9%) had diabetes mellitus and 94 (34.9%) had hyperlipidemia.

Patients with more extensive coronary artery disease were significantly older. Higher prevalence of smoking, hypertension, diabetes, and hyperlipidemia was observed in triple-vessel disease. BMI and sex distribution showed no statistically significant differences (borderline for sex). These findings are illustrated in Table 1. Differences between groups were assessed using one-way ANOVA for continuous variables (Age, BMI) and the chi-square test for categorical variables (Sex, Smoking, Hypertension, Diabetes, Hyperlipidemia).

### 3.2. Coronary Artery Disease Extension

**Figure 2:**
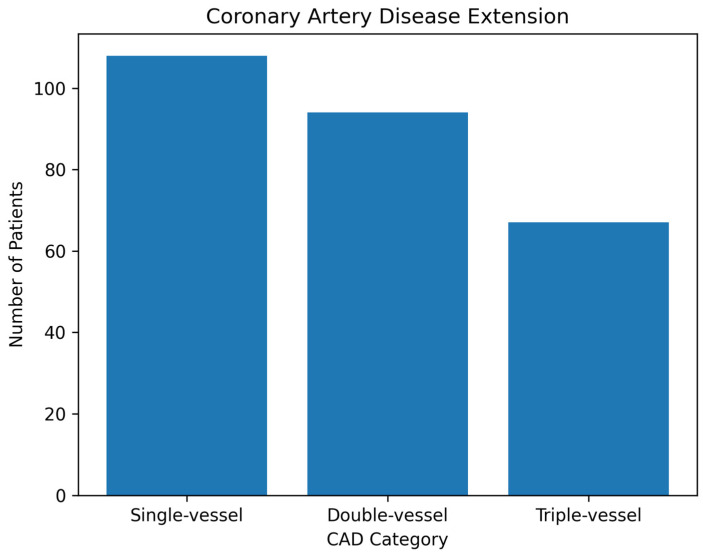
Distribution of coronary artery disease extension. Bar chart illustrating the number of patients with single-, double- and triple-vessel coronary artery disease as determined by coronary angiography.

Coronary angiography revealed single-vessel disease in 108 patients (40.1%), double-vessel disease in 94 patients (34.9%) and triple-vessel disease in 67 patients (24.9%). The distribution of CAD extension categories is illustrated in Figure 2.

### 3.3. Daily Preadmission Effort and CAD Severity

Daily preadmission effort, assessed using the 1–10 self-reported scale, showed a marked inverse relationship with CAD severity. Mean effort scores progressively decreased with increasing CAD extension:

- Single-vessel disease: 7.44.
- Double-vessel disease: 4.93.
- Triple-vessel disease: 3.69.

Comparison across CAD categories using the Kruskal–Wallis test demonstrated a highly significant difference in daily effort scores (H = 85.82, *p* < 0.0001).

**Figure 3:**
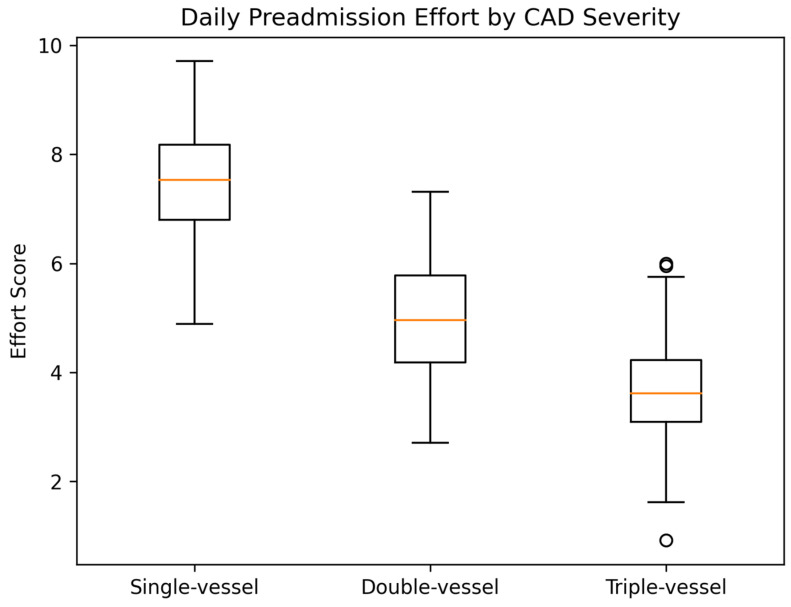
Daily preadmission effort according to coronary artery disease extension.

The distribution of daily preadmission effort across CAD severity categories is shown in Figure 3.

Box-and-whisker plots showing daily preadmission effort scores across single-, double- and triple-vessel disease categories. Median values and interquartile ranges are displayed. Differences between groups were assessed using the Kruskal–Wallis test.

### 3.4. Stratified Analysis by Age

The association between daily preadmission effort and CAD extent remained consistent across age strata. In both younger (≤median age) and older (>median age) patients, lower effort levels were associated with more extensive CAD, whereas higher effort levels were predominantly observed in patients with single-vessel disease (both *p* < 0.001). The results are presented in Table 2 and Table 3.

### 3.5. Ordinal Logistic Regression Analysis

To identify independent predictors of coronary artery disease extension, an ordinal logistic regression (proportional odds model) was performed, with CAD severity (single-, double-, triple-vessel disease) treated as an ordered outcome.

Increasing age was independently associated with greater CAD severity (OR 1.07 per year, 95% CI 1.04–1.10, *p* < 0.0001). Hypertension (OR 2.48, 95% CI 1.17–5.29, *p* = 0.018), diabetes mellitus (OR 3.95, 95% CI 1.64–9.51, *p* = 0.002) and hyperlipidemia (OR 2.63, 95% CI 1.22–5.67, *p* = 0.013) were also significant independent predictors of more extensive CAD.

Sex and smoking status showed positive but non-significant associations with CAD severity, while BMI was not significantly associated with disease extension.

Importantly, daily preadmission effort was strongly and independently protective. Each one-point increase in the effort score was associated with a 46% reduction in the odds of being in a higher CAD severity category (OR 0.54, 95% CI 0.45–0.64, *p* < 0.0001).

The results of the ordinal logistic regression analysis are presented in Table 4.

### 3.6. Sensitivity Analysis

When analyzed as a categorical variable, the daily preadmission effort score remained strongly associated with CAD extent. Patients with low effort were predominantly represented in the triple-vessel group, whereas those with high effort were mainly observed in the single-vessel group. This pattern was consistent with a graded inverse relationship between effort level and disease severity (*p* < 0.001). These results are shown in Table 5.

### 3.7. Correlation Analysis

**Figure 4:**
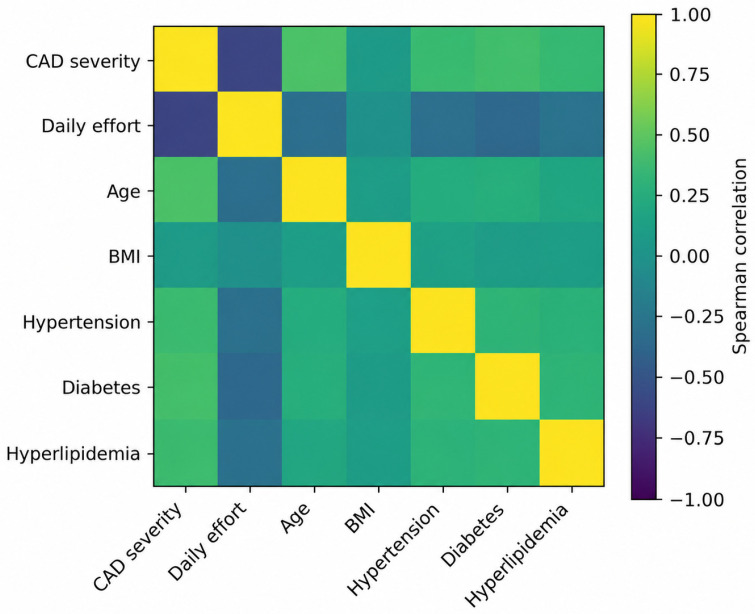
Correlation matrix of clinical variables, daily preadmission effort and coronary artery disease severity.

Exploratory correlation analysis demonstrated a strong inverse association between daily preadmission effort and CAD severity, as well as positive correlations between CAD severity and age, hypertension, diabetes and hyperlipidemia. These relationships are illustrated in Figure 4.

Heatmap displaying Spearman correlation coefficients between coronary artery disease severity, daily preadmission effort, age, body mass index and traditional cardiovascular risk factors. Color intensity reflects the strength and direction of correlations.

### 3.8. Summary of Key Findings

In this cohort of patients presenting with myocardial infarction, greater habitual daily physical activity prior to admission was strongly associated with less extensive coronary artery disease. The inverse relationship between daily preadmission effort and CAD severity was consistent across unadjusted and multivariable analyses and remained robust after adjustment for traditional cardiovascular risk factors.

## 4. Discussion

### 4.1. Principal Findings

In this prospective observational study of patients presenting with MI, we identified a strong and independent inverse association between habitual daily physical activity prior to admission and the angiographic extent of coronary artery disease. Higher daily preadmission effort was consistently associated with lower CAD severity, demonstrating a clear gradient across single-, double- and triple-vessel disease categories. Importantly, this association remained robust after adjustment for traditional cardiovascular risk factors, including age, hypertension, diabetes mellitus and hyperlipidemia.

Using an ordinal logistic regression framework allowed us to model CAD severity as a graded outcome, reflecting the clinical reality that coronary disease burden exists along a continuum rather than as isolated categories. Within this model, each one-point increase in daily preadmission effort was associated with a substantial reduction in the odds of more extensive CAD, underscoring the potential clinical relevance of habitual everyday activity in relation to coronary atherosclerotic burden. This magnitude of association is substantial and suggests that even modest differences in habitual daily activity may translate into clinically meaningful differences in coronary atherosclerotic burden. Compared to traditional risk factors, the strength of the association observed for daily effort is comparable to or greater than that of several established predictors, highlighting its potential relevance as a marker of underlying disease severity.

Age, hypertension, diabetes mellitus and hyperlipidemia showed strong independent associations with CAD severity. The magnitude of these effects highlights the cumulative impact of metabolic and vascular risk factors on atherosclerotic burden. For example, diabetes demonstrated the highest odds ratio, consistent with its known role in accelerating diffuse coronary atherosclerosis.

Sex and smoking status showed non-significant trends, which may reflect limited statistical power or population-specific characteristics. BMI was not associated with CAD extension, potentially reflecting the limitations of BMI as a marker of metabolic risk compared to measures such as visceral adiposity.

Although the physical activity scale used in this study was not formally validated, its ordinal structure may still capture meaningful gradients of habitual activity, similar to simplified activity indices used in large epidemiological cohorts. The strong and consistent dose–response relationship observed supports its construct validity in this context.

An important consideration in interpreting these findings is the possibility of reverse causality. Patients with more advanced coronary disease may have reduced their activity levels due to subclinical or overt symptoms prior to infarction. Therefore, habitual activity may function both as a protective factor and as a marker of underlying disease burden.

### 4.2. Comparison with Previous Studies

The protective association between physical activity and coronary artery disease is well-established. Large epidemiological studies and meta-analyses have consistently demonstrated that higher levels of physical activity are associated with reduced incidence of coronary events, cardiovascular mortality and all-cause mortality [22,23,24]. A 2023 dose–response meta-analysis of 94 cohorts with over 30 million participants found that achieving 8.75 marginal metabolic equivalent task-hours per week (equivalent to 150 min/week of moderate-to-vigorous activity) was associated with a 31% reduction in all-cause mortality and a 29% reduction in cardiovascular disease mortality [25]. The PURE study of 130,000 individuals from 17 countries demonstrated that meeting physical activity guidelines was associated with a 22% reduction in mortality and a 20% reduction in major cardiovascular disease [26]. However, most prior investigations have focused on structured exercise or leisure-time physical activity, often assessed through detailed questionnaires that are impractical in acute clinical settings.

Our findings extend this body of evidence by focusing on habitual daily activity, encompassing occupational, domestic and incidental movement. This broader construct may better reflect real-world behavior and cumulative physiological adaptation than formal exercise alone. Few studies have specifically examined the relationship between preadmission daily activity and angiographic CAD severity in patients with acute MI. A 2019 coronary computed tomography angiography study by Feuchtner et al. found that regular moderate-to-high endurance exercise (≥3 times/week, ≥1 h) was associated with lower prevalence of >50% stenosis (13.2% vs. higher rates in inactive individuals), reduced stenosis severity scores and lower total and non-calcified plaque burden [27]. Similarly, Hambrecht et al. demonstrated in a landmark 1993 randomized trial that patients expending approximately 2200 kcal/week in leisure-time physical activity showed regression of coronary lesions, whereas those expending 1022 kcal/week experienced disease progression [28]. More recently, a 2024 study of 9772 patients undergoing coronary CT angiography found a stepwise inverse relationship between self-reported exercise activity and mortality, with patients reporting no activity having a three-fold higher mortality risk compared to those with high activity levels, even after adjustment for stenosis severity [29]. Notably, the risk of all-cause mortality was similar among patients with obstructive stenosis who engaged in high exercise versus those with no coronary stenosis but no exercise activity, suggesting that physical activity may substantially modify the prognostic impact of anatomic disease burden. However, these studies primarily assessed leisure-time or structured exercise rather than comprehensive habitual daily activity including occupational and domestic domains. By demonstrating a strong inverse association with multivessel disease using a simple assessment of total habitual daily effort, our study adds novel evidence that habitual daily activity may be linked not only to the occurrence of CAD but also to its anatomical extent at the time of acute MI presentation.

However, not all studies consistently demonstrate the protective effect of high levels of physical activity on coronary atherosclerosis or post-infarction outcomes. A recent study by Feuchtner et al. [30] using coronary CT angiography reported that very high levels of exercise may be associated with a distinct atherosclerotic profile, including increased coronary calcification, without clear reduction in overall plaque burden. Similarly, experimental data by Dias et al. [31] showed that prior aerobic exercise training did not confer consistent cardioprotection during early post-infarction remodeling in animal models, regardless of exercise volume.

These discrepancies may be explained by differences in study populations (stable CAD vs. acute MI), types and intensity of physical activity (structured exercise vs. habitual daily activity), and outcome measures (plaque composition vs. angiographic extent). Additionally, very high-intensity or prolonged exercise may induce distinct vascular adaptations that differ from those associated with moderate habitual activity. In our study, we assessed global daily effort rather than structured or extreme exercise, which may better reflect physiologically sustainable activity patterns associated with cardiovascular benefit.

To our knowledge, this is one of the few studies to evaluate the association between global habitual daily activity—rather than structured exercise—and angiographic coronary artery disease extent in an acute myocardial infarction population.

### 4.3. Potential Pathophysiological Mechanisms

Several biological mechanisms may explain the observed association between higher habitual activity and less extensive coronary disease. Regular physical activity improves endothelial function through enhanced nitric oxide bioavailability, leading to improved vasodilation and reduced vascular stiffness. Meta-analyses demonstrate that exercise training increases brachial artery flow-mediated dilation by approximately 2–4%, with each 1% increase associated with a 13% reduction in future cardiovascular events. Physical activity also reduces systemic inflammation, improves insulin sensitivity, lowers blood pressure and favorably modifies lipid profiles, which are all key contributors to atherosclerotic progression.

Moreover, habitual activity may promote plaque stability and limit diffuse atherosclerosis by reducing oxidative stress and improving vascular remodeling. Long-term aerobic exercise training induces shear stress-mediated arterial remodeling that results in larger conduit artery sizes and reduced wall thickness, markedly increasing luminal reserve and reducing the probability of flow-limiting stenosis. Two randomized trials using intravascular ultrasound assessed the impact of intensive exercise on plaque regression; in one trial’s post hoc analysis, patients who walked ≥7000 steps per day had greater plaque regression compared with those who walked <7000 steps per day (−12.5% vs. <3.6%) [32]. A 2015 study found that aerobic exercise-induced beneficial changes in coronary atherosclerosis via reduced necrotic core volume (−4.94 mm^3^ in stable CAD patients), with clinical presentation being the strongest predictor of exercise-induced plaque stabilization [33]. Cross-sectional studies using coronary CT angiography demonstrate that athletes have higher calcific plaque volume whereas sedentary individuals have greater mixed plaque morphologies, which are of higher risk for coronary events. However, a 2025 review noted that recent evidence challenges the hypothesis that increased coronary artery calcification in athletes represents plaque stabilization, as athletes with elevated coronary artery calcium show increased cardiovascular risk, though their higher fitness levels partially mitigate this risk [34]. Exercise training has been shown to reduce necrotic core volume and stabilize plaque morphology through the restoration of β2-adrenergic receptor signaling and the inhibition of inflammatory pathways [35]. Over time, these cumulative effects may result in less extensive coronary involvement, even in individuals who ultimately experience myocardial infarction. While our observational design does not allow causal inference, the strength, consistency and dose–response nature of the association support its biological plausibility.

### 4.4. Clinical Implications

The findings of this study have several potential clinical implications. First, simple assessment of habitual daily activity at hospital admission may provide useful information regarding underlying coronary disease burden [36,37,38]. Unlike complex physical activity questionnaires, a brief self-rated effort scale is feasible in acute-care settings and may complement traditional risk stratification.

Second, our results emphasize the importance of promoting everyday physical activity, not only structured exercise programs [39,40]. For many patients, particularly older adults or those with comorbidities, maintaining higher levels of daily movement through occupational, household, or routine activities may be more achievable and sustainable than formal exercise regimens. However, it is important to note that recent evidence suggests domain-specific effects of physical activity. A 2025 NHANES analysis found that leisure-time and transportation-related physical activity were associated with 22% and 40% lower odds of CVD, respectively, whereas occupational physical activity showed no significant protective effect [41]. Among MI survivors specifically, maintaining high physical activity or increasing activity from before to after MI was associated with 27–39% lower mortality risk and 22–37% lower risk of recurrent nonfatal cardiovascular events. Recent meta-analyses of daily step counts provide additional support for dose–response relationships, with risk reductions for cardiovascular events becoming statistically significant at approximately 2735 steps/day and reaching maximal benefit at around 7000–8800 steps/day [42,43]. Encouraging incremental increases in daily effort could therefore represent a pragmatic approach to cardiovascular prevention and secondary prevention after myocardial infarction.

### 4.5. Strengths and Limitations

This study has several notable strengths. The prospective design and inclusion of consecutive patients reduce selection bias. Coronary artery disease severity was assessed using coronary angiography, providing an objective and clinically relevant outcome measure. The use of an ordinal regression model appropriately reflected the ordered nature of CAD severity and allowed for efficient estimation of associations across disease categories. Additionally, adjustment for multiple established cardiovascular risk factors strengthens the validity of the observed independent association between daily effort and CAD extension.

However, several limitations should be acknowledged. A major limitation of this study is the potential for reverse causality. Given that physical activity was assessed during the four weeks preceding myocardial infarction, it is plausible that some patients experienced prodromal symptoms of coronary insufficiency, such as angina or dyspnea, which may have limited their habitual physical activity. This is particularly relevant in patients with more advanced coronary artery disease, in whom symptom burden is likely higher. Therefore, the observed inverse association between daily preadmission effort and CAD severity may partially reflect reduced activity as a consequence of underlying disease rather than a causal protective effect of physical activity.

Although we attempted to minimize this bias by excluding patients with severe conditions limiting physical activity and by assessing activity shortly after admission, we did not systematically collect data on pre-infarction symptoms. Future studies should incorporate detailed assessment of symptom burden prior to myocardial infarction to better disentangle causality.

Daily preadmission effort was assessed using a self-reported scale, which is subject to recall bias and subjective interpretation. Although the scale was designed to capture habitual activity in a pragmatic manner, objective measurements such as accelerometry would provide greater precision. Importantly, the physical activity scale used in this study was not formally validated, which may limit measurement accuracy and reproducibility. The reliance on self-reported data introduces potential recall bias and subjective interpretation. Additionally, the single-center design may limit external validity. The observational nature of the study precludes causal inference and residual confounding by unmeasured factors, such as diet, socioeconomic status or genetic predisposition, which cannot be excluded. Furthermore, this was a single-center study, which may limit the generalizability of the findings to other populations or healthcare settings.

### 4.6. Future Direction

Future research should aim to validate these findings in larger, multicenter cohorts and across diverse populations. Studies incorporating objective measures of physical activity, such as wearable devices, would help confirm and refine the observed associations. Longitudinal investigations are also needed to determine whether increases in habitual daily activity after myocardial infarction can influence the progression of coronary artery disease, reduce recurrent ischemic events, or improve long-term outcomes. Interventional studies targeting everyday physical activity, rather than structured exercise alone, may provide valuable insights into practical prevention strategies.

## 5. Conclusions

In this prospective observational study, higher habitual daily physical activity prior to myocardial infarction was associated with less extensive coronary artery disease. This relationship demonstrates a clear graded pattern across increasing CAD severity and persists after adjustment for traditional cardiovascular risk factors. However, these findings should be interpreted cautiously given the observational design and use of a non-validated self-reported measure. Further studies are needed to confirm these associations.

## Figures and Tables

**Table 1:** Baseline characteristics by CAD extension.

Variable	Single-Vessel	Double-Vessel	Triple-Vessel	*p*-Value
Age	55.1 ± 14.8	59.5 ± 14.0	64.4 ± 12.6	<0.001
BMI	27.0 ± 4.2	26.5 ± 4.0	28.1 ± 4.2	0.064
Sex (male)	40.7%	41.5%	58.2%	0.051
Smoking	38.9%	46.8%	59.7%	0.027
Hypertension	28.7%	38.3%	47.8%	0.037
Diabetes	16.7%	30.9%	37.3%	0.006
Hyperlipidemia	26.9%	37.2%	49.3%	0.011

Notes: Data are mean ± SD or %. ANOVA for continuous variables and chi-square for categorical variables. *p* < 0.05 significant.

**Table 2:** Distribution of daily preadmission effort categories across CAD extent in patients > median age.

Effort Category	Single-Vessel	Double-Vessel	Triple-Vessel
Low (1–4)	4 (8.9%)	16 (34.0%)	24 (57.1%)
Medium (5–7)	8 (17.8%)	17 (36.2%)	15 (35.7%)
High (8–10)	33 (73.3%)	14 (29.8%)	3 (7.1%)

Notes: Data are presented as *n* (%), with percentages calculated within each CAD group. Effort categories were defined as low (1–4), medium (5–7), and high (8–10). Differences were assessed using the chi-square test.

**Table 3:** Distribution of daily preadmission effort categories across CAD extent in patients ≤ median age.

Effort Category	Single-Vessel	Double-Vessel	Triple-Vessel
Low (1–4)	9 (14.3%)	25 (53.2%)	20 (80.0%)
Medium (5–7)	25 (39.7%)	20 (42.6%)	5 (20.0%)
High (8–10)	29 (46.0%)	2 (4.3%)	0 (0.0%)

Notes: Data are presented as *n* (%), with percentages calculated within each CAD group. Effort categories were defined as low (1–4), medium (5–7), and high (8–10). Differences were assessed using the chi-square test.

**Table 4:** Ordinal logistic regression analysis for predictors of coronary artery disease extension. Results of the multivariable proportional odds model assessing independent predictors of increasing CAD severity.

Predictor	Odds Ratio	95% Confidence Interval	*p*-Value
Age (per year)	1.07	1.04–1.10	<0.0001
Male sex	1.65	0.79–3.46	0.184
Smoking	1.74	0.85–3.55	0.130
Hypertension	2.48	1.17–5.29	0.018
Diabetes mellitus	3.95	1.64–9.51	0.002
Hyperlipidemia	2.63	1.22–5.67	0.013
Body mass index (kg/m^2^)	0.99	0.91–1.08	0.814
Daily preadmission effort (per point)	0.54	0.45–0.64	<0.0001

Note: Odds ratios represent the odds of being in a higher coronary artery disease severity category. All variables were entered simultaneously into the model. Statistical significance was defined as *p* < 0.05.

**Table 5:** Distribution of daily preadmission effort categories across CAD) extent.

Effort Category	Single-Vessel	Double-Vessel	Triple-Vessel
Low (1–4)	13 (12.0%)	41 (43.6%)	44 (65.7%)
Medium (5–7)	33 (30.6%)	37 (39.4%)	20 (29.9%)
High (8–10)	62 (57.4%)	16 (17.0%)	3 (4.5%)

Note: Data are presented as *n* (%), with percentages calculated within each CAD group. The daily preadmission effort score was categorized into tertiles as follows: low (1–4), medium (5–7), and high (8–10). Differences between groups were assessed using the chi-square test. A *p*-value < 0.05 was considered statistically significant. A significant association was observed (*p* < 0.001).

## Data Availability

The raw data supporting the conclusions of this article will be made available by the authors upon request. The original contributions presented in this study are included in the article. Further inquiries can be directed to the corresponding author.

## Supplementary Materials

The following supporting information can be downloaded at https://www.mdpi.com/article/10.3390/jcm15103814/s1, Supplementary Materials S1: Extended Questionnaire on Daily Physical Activity Prior to Admission.
