# Supplementary material for: Self-Reported Habitual Daily Physical Activity as an Independent Predictor of Coronary Artery Disease Extension in Patients with Myocardial Infarction: A Prospective Observational Study
Source: J Clin Med. 2026 May 15;15(10):3814. doi: 10.3390/jcm15103814 (PMC13207773; doi:10.3390/jcm15103814)
Supplement: Supplementary file 1 [file jcm-15-03814-s001.zip › jcm-4283167-supplementary.pdf]

**Supplementary Materials S1. Extended Questionnaire on Daily Physical Activity Prior to Admission**

Participant Name: ___________________________

Date: ____ / ____ / ________

## Instructions

Please answer the following questions regarding your level of physical activity during the last 4 weeks prior to your current hospital admission. Physical activity includes all forms of movement such as occupational activity, walking, household tasks, recreational activities, and exercise. Please select the option that best describes your usual level of activity.

## Section 1. Basic Daily Activity

1. How often do you walk for at least 15 minutes?
☐ Never ☐ Rarely ☐ Occasionally ☐ Frequently ☐ Daily

2. How often do you climb stairs instead of using an elevator?
☐ Never ☐ Rarely ☐ Occasionally ☐ Frequently ☐ Daily

3. How often do you perform household activities (cleaning, cooking, shopping)?
☐ Never ☐ Rarely ☐ Occasionally ☐ Frequently ☐ Daily

## Section 2. Occupational Activity

4. Does your work involve physical movement or effort?
☐ Very little ☐ Little ☐ Moderate ☐ High ☐ Very high

5. During your working hours, how much time do you spend sitting?
☐ Very little ☐ Little ☐ Moderate ☐ High ☐ Very high

## Section 3. Recreational Activity

6. Do you engage in regular sports or exercise (e.g., running, cycling, swimming, gym)?
☐ Never ☐ Rarely ☐ Occasionally ☐ Frequently ☐ Daily

7. Do you participate in recreational activities involving movement (e.g., gardening, walking, active hobbies)?
☐ Never ☐ Rarely ☐ Occasionally ☐ Frequently ☐ Daily

## Section 4. Perceived Effort and Functional Capacity

8. Do you feel fatigued after your usual daily activities?
☐ Never ☐ Rarely ☐ Occasionally ☐ Frequently ☐ Always

9. Do you experience shortness of breath during normal walking or climbing stairs?
☐ Never ☐ Rarely ☐ Occasionally ☐ Frequently ☐ Always

10. Do you consider your energy level sufficient for daily activities?
☐ Never ☐ Rarely ☐ Occasionally ☐ Frequently ☐ Always

## Section 5. Global Effort Score

Please rate your overall level of daily physical effort during the last 4 weeks on a scale from 1 to 10:

- 1–2: predominantly sedentary behavior
- 3–4: light daily activity (minimal walking, low occupational effort)
- 5–6: moderate activity (regular walking, light physical work)
- 7–8: high activity (physically demanding daily routines)
- 9–10: very high activity (intense manual labor or sustained exertion)

Score: ____ / 10

This questionnaire and the associated global effort scale were developed by the investigators for the purpose of this study and have not been formally validated.
